# CHRONIC PAIN AND ASSOCIATED RISK OF COGNITIVE IMPAIRMENT AMONG MIDDLE-AGED AND OLDER ADULTS

**DOI:** 10.1093/geroni/igz038.2637

**Published:** 2019-11-08

**Authors:** Yi-Han Hu, Hsien-Chang Lin

**Affiliations:** Indiana University Bloomington, Bloomington, Indiana, United States

## Abstract

Chronic non-cancer pain (CNCP) is an emerging health issue among the older population. Not only did the CNCP prevalence increase gradually in past decades, but also it may cause difficulties in cognitive processing and social and emotional functioning. However, evidence for the associations between CNCP and incident mild cognitive impairment (MCI) and Alzheimer’s disease and related dementias (ADRDs) is inconsistent and insufficient. Using the administrative claims data from health insurance companies from January 2007 to December 2017, this prospective cohort study investigated the impact of CNCP on the risks of developing MCI and ADRDs among adults aged 50 and older. To reduce potential selection bias, the propensity-score matched cohort design was applied for selecting comparable CNCP and non-CNCP patients at the beginning of the follow-up. Time-dependent Cox proportional-hazards regression models were conducted to estimate the hazard ratios (HRs) of incident MCI/ADRDs, adjusting for baseline sociodemographics and time-dependent medical conditions. Of 236,782 patients with/without CNCP, 342 individuals (0.14%) developed MCI and 1,183 patients (5.0%) had been diagnosed with one type of ADRDs during the follow-up. After adjusting confounders, CNCP patients had a 42% increased MCI risk (HR=1.42; 95% CI=1.14-1.76) and a 20% increased ADRDs risk (HR=1.20; 95% CI=1.07-1.34) relative to non-CNCP patients. Our findings indicate that CNCP is associated with incidences of MCI and ADRDs. Early diagnosis of CNCP and CNCP management may prevent cognitive impairment among middle-aged and older adults. Future studies are warranted to explore the potential effects of pain treatments on restoring cognitive function of CNCP patients.

